# A Rare Case of Non-Neurological Ptosis

**DOI:** 10.7759/cureus.49731

**Published:** 2023-11-30

**Authors:** Bander Otayf, Duaa Baarmah

**Affiliations:** 1 Division of Neurology, Department of Paediatrics, King Abdullah Specialized Children’s Hospital, King Abdulaziz Medical City, Riyadh, SAU; 2 Department of Paediatrics, King Abdullah bin Abdulaziz University Hospital, Princess Nourah bint Abdulrahman University, Riyadh, SAU

**Keywords:** fatigable ptosis, curtain signs, giant papillary, blepharoptosis, vernal keratoconjunctivitis (vkc)

## Abstract

Ptosis in pediatrics is commonly attributed to neurological causes. Rarely, chronic inflammation of the upper eyelid and the formation of giant papillary conjunctivitis due to vernal keratoconjunctivitis (VKC) may lead to ptosis. In this case report, we present an eight-year-old girl with conjunctivitis who was referred to a pediatric neurology service for evaluation of ptosis. She presented with progressive left-eye ptosis while experiencing allergic conjunctivitis. Her neurological exam showed non-fatigable ptosis with a negative curtain sign. The rest of the neurology examination was normal. She tested negative for anti-MuSK and anti-Ach. The orbital MRI was unremarkable. Further detailed examination by an ophthalmologist showed severe VKC with a giant papillary formation that had led to mechanical ptosis.

## Introduction

The drooping of the upper eyelid margin on the primary gaze is called blepharoptosis, which results in covering part of the eye and narrowing the palpebral opening [1]. The causes of blepharoptosis can be classified according to its pathogenesis, such as myogenic, neurogenic, aponeurogenic, or mechanical [2].

In general, ptosis in pediatrics is commonly caused by neurological factors, but acquired causes are commonly due to aponeurogenic blepharoptosis and associated with disinsertion of the levator aponeurosis from its distal insertion in the eyelid, though the levator function is good [3,4]. In the elderly, aponeurogenic blepharoptosis is commonly caused by involutional disorders, but in the younger population, it is most commonly due to contact lens wear [3]. It can also be associated with other conditions, such as ocular inflammation, intraocular surgery, postoperative edema, and topical steroid use [3]. However, acquired ptosis can occur in younger adults without a history of wearing contact lenses, intraocular surgery, or trauma [3].

Rare cases have shown mild to moderate ptosis associated with vernal keratoconjunctivitis (VKC) [3]. Prolonged severe VKC leads to chronic inflammation of the upper eyelid, the formation of giant papillae conjunctivitis, and persistent eyelid rubbing, which may be responsible for the development of ptosis [3]. In this case report, we aim to present a rare case of ptosis in a patient with VKC.

## Case presentation

**Figure 1:**
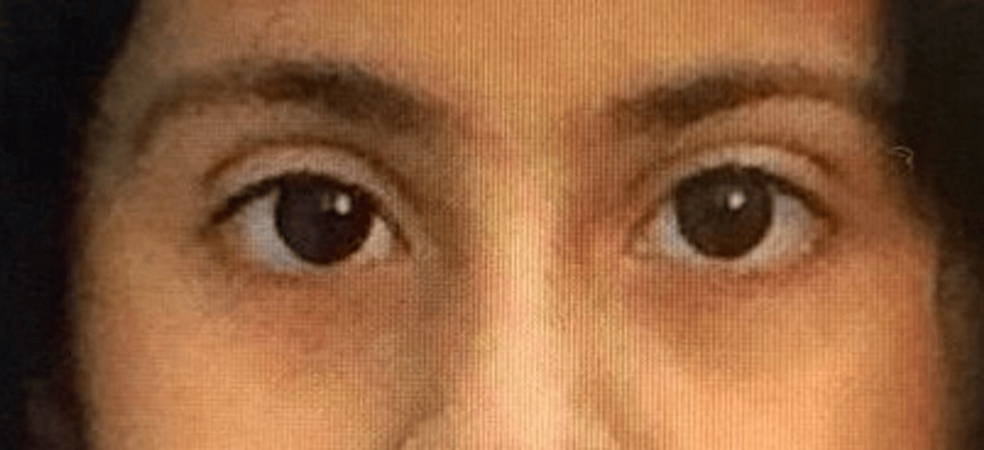
Normal eyelid position before the disease started

An eight-year-old girl is known to have VKC and wears glasses to correct refractive errors. She was referred to the pediatric neurology service for an evaluation of bilateral ptosis. She presented with progressive bilateral eyelid ptosis three weeks ago. The ptosis was intermittent, more on the left side, lasting for days, and was associated with itchiness and tearing. It was not associated with diurnal variation, headaches, double vision, weakness, or fatigue. The patient has not had a fever, an upper respiratory tract infection, significant weight changes, or changes in appetite. She has a history of an atrial septal defect, which resolved without treatment, and experienced neck pain a year ago due to a vitamin D deficiency. This was successfully treated with a vitamin D supplement. There is no history of similar conditions, surgeries, or blood transfusions. She was delivered through a spontaneous, normal vaginal delivery at full term and did not require neonatal intensive care unit admission. Currently, she is performing well in second grade at school. The family history of the patient indicates that the parents are first-degree relatives. The father is 39 years old with diabetes, the mother is 36 years old with hypothyroidism, and there are four siblings, three of whom have refractive errors. The patient is under the care of an ophthalmologist and is prescribed two medications (olopatadine and ocular lubricant) for the treatment of VKC. Figure 1 shows the normal eyelid position of the patient before the disease started.

**Figure 2:**
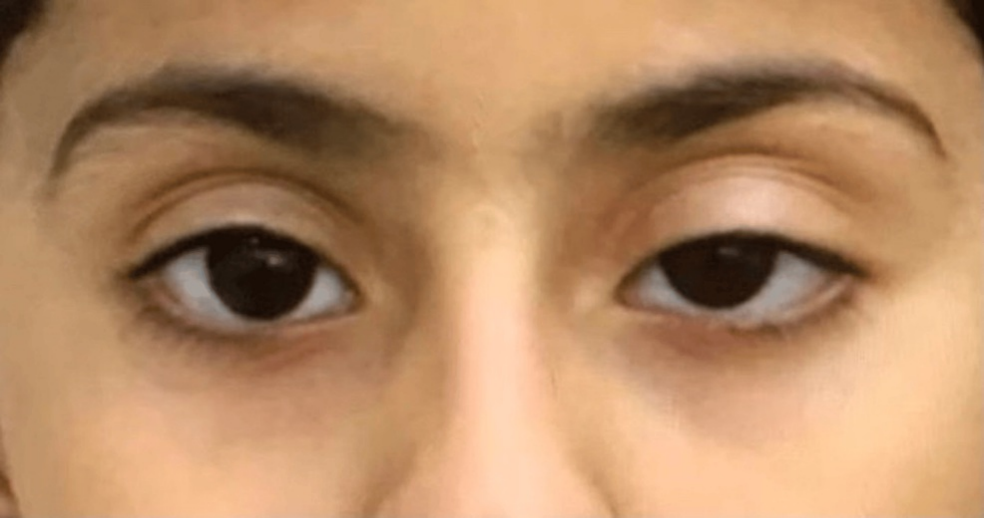
Bilateral mild ptosis more on the left side

During the examination, the patient presented as well, alert, and oriented. Her speech was clear and fluent, and she displayed no dysmorphic features. She was stable in terms of vital signs and hemodynamics, with normal anthropometric measurements. The eye examination revealed bilateral congested eyes, with slightly more prominent bilateral ptosis in the left eye (Figure 2). Both the curtain sign and fatigability test yielded negative results. Pupillary responses were equal and reactive to light in both eyes, and full extraocular movements were observed. There was no evidence of facial asymmetry, and sensation was intact in all three divisions bilaterally. Hearing function was also normal. The motor examination demonstrated normal tone, power, and reflexes in all limbs. Gait and coordination were unimpaired, and there were no discernible skin stigmata.

The autoimmune serology showed negative results for both muscle-specific receptor tyrosinase antibodies (anti-MuSK) and acetylcholine receptor antibodies (anti-Ach) (Table 1). The orbit MRI yielded unremarkable results.

**Table 1:** Autoimmune serology Anti-MuSK: muscle-specific receptor tyrosinase antibodies, Anti-Ach: acetylcholine receptor antibodies

Antibodies	Result	Normal range
Anti-MuSK	<0.02 nmol/l	<0.05 nmol/l
Anti-Ach	<0.10 nmol/l	<0.25 nmol/l

The patient was referred to a pediatric ophthalmologist for a thorough examination, which revealed severe VKC and a giant papillary formation causing mechanical ptosis. Treatment with olopatadine and ocular lubricant was initiated. The family received instructions regarding concerning signs and was advised to avoid allergic triggers for the eyes.

After one month of treatment, the ptosis showed a noticeable improvement. The patient was subsequently scheduled for a follow-up appointment at the ophthalmology clinic to monitor her allergic conjunctivitis. During a follow-up visit one year later, it was observed that activation of her allergic conjunctivitis would result in a recurrence of ptosis.

## Discussion

In this case, we draw attention to the uncommon occurrence of ptosis in a patient with VKC. While pediatric ptosis is most frequently associated with neurological causes, it can occasionally manifest as a severe complication in VKC patients due to chronic inflammation of the upper eyelid, leading to the formation of giant papillary conjunctivitis and persistent eyelid rubbing [3]. Neurological ptosis typically presents with features of neurological dysfunction, including visual complaints, third cranial nerve palsy, and Horner's syndrome [1]. Although, based on the patient’s history, neurological causes were deemed less likely, further neurology investigations were pursued as non-neurologic causes are unusual in the pediatric age group [1]. Treatment with olopatadine led to a reduction in the symptoms of allergic conjunctivitis [5]. We highlight the significance of comprehensive history-taking, physical examination, and diagnostic tests in establishing a diagnosis. This is evident in this case, where the patient developed ptosis alongside features of conjunctivitis with normal neurological findings. Furthermore, treatment with olopatadine and ocular lubricant proved to be effective in managing VKC and improving ptosis. It is, however, imperative to educate the family about the importance of averting serious complications of VKC by avoiding allergens that trigger eye reactions, as ptosis may recur with the activation of allergic conjunctivitis.

## Conclusions

This case highlights the correlation between VKC and ptosis in a pediatric patient. Effective management through medication and patient education can result in symptom alleviation. However, the reappearance of ptosis alongside allergic conjunctivitis serves as a reminder of the necessity for continuous vigilance and avoidance of triggers to avert complications. Regular follow-up appointments with ophthalmology are crucial for monitoring the patient’s progress. Prompt recognition of symptoms by pediatricians is vital to ensure timely referral to the appropriate healthcare service.
